# Assessing retention in care after 12 months of the Pediatric Development Clinic implementation in rural Rwanda: a retrospective cohort study

**DOI:** 10.1186/s12887-018-1007-0

**Published:** 2018-02-16

**Authors:** Scheilla Bayitondere, Francois Biziyaremye, Catherine M. Kirk, Hema Magge, Katrina Hann, Kim Wilson, Christine Mutaganzwa, Eric Ngabireyimana, Fulgence Nkikabahizi, Evelyne Shema, David B. Tugizimana, Ann C. Miller

**Affiliations:** 1 Ministry of Health, Rwinkwavu District Hospital, Rwinkwavu, Rwanda; 2 Partners In Health/Inshuti Mu Buzima, Rwinkwavu, Rwanda; 3 Boston Children’s Hospital, Boston, MA USA; 4 Brigham and Women’s Hospital, Boston, MA USA; 5 Partners In Health Sierra Leone, Freetown, Sierra Leone; 6 Harvard Medical School, Boston, MA USA

**Keywords:** loss to follow-up, high-risk infants, kangaroo mother care, social support, early childhood development, prematurity, Rwanda, Sub-Saharan Africa

## Abstract

**Background:**

In Africa, a high proportion of children are at risk for developmental delay. Early interventions are known to improve outcomes, but they are not routinely available. The Rwandan Ministry of Health with Partners In Health/Inshuti Mu Buzima created the Pediatric Development Clinic (PDC) model for providing interdisciplinary developmental care for high-risk infants in rural settings. As retention for chronic care has proven challenging in many settings, this study assesses factors related to retention to care after 12 months of clinic enrollment.

**Methods:**

This study describes a retrospective cohort of children enrolled for 12 months in the PDC program in Southern Kayonza district between April 2014–March 2015. We reviewed routinely collected data from electronic medical records and patient charts. We described patient characteristics and the proportion of patients retained, died, transferred out or lost to follow up (LTFU) at 12 months. We used Fisher’s exact test and multivariable logistic regression to identify factors associated with retention in care.

**Results:**

228 children enrolled in PDC from 1 April 2014–31 March 2015, with prematurity/low birth weight (62.2%) and hypoxic ischemic encephalopathy (34.5%) as the most frequent referral diagnoses. 64.5% of children were retained in care and 32.5% were LTFU after 12 months. In the unadjusted analysis, we found male sex (*p* = 0.189), having more children at home (*p* = 0.027), health facility of first visit (*p* = 0.006), having a PDC in the nearest health facility (*p* = 0.136), referral in second six months of PDC operation (*p* = 0.006), and social support to be associated (100%, *p* < 0.001) with retention after 12 months. In adjusted analysis, referral in second six months of PDC operation (Odds Ratio (OR) 2.56, 95% CI 1.36, 4.80) was associated with increased retention, and being diagnosed with more complex conditions (trisomy 21, cleft lip/palate, hydrocephalus, other developmental delay) was associated with LTFU (OR 0.34, 95% CI 0.15, 0.76). As 100% of those receiving social support were retained in care, this was not able to be assessed in adjusted analysis.

**Conclusions:**

PDC retention in care is encouraging. Provision of social assistance and decentralization of the program are major components of the delivery of services related to retention in care.

## Background

In low- and middle-income countries, almost 250 million children under five years of age are estimated to be at risk for delay in intellectual, physical, psychological, or social abilities [1, 2]. Children born preterm, at low birth weight, or with other medical conditions at birth are at even greater risk for impaired growth and development [3, 4]. Early childhood interventions during the first years of life can play a major role in improving the future outcome for the child’s development [5]. In addition, children who have higher participation in early intervention programs designed for at-risk children report greater benefits and longer lasting effects than those with less participation [6].

In an attempt to meet the child survival fourth Millennium Development Goal, many countries, including Rwanda, made significant progress in terms of improvement of child health, and subsequently, the reduction of child mortality [7]. With strong leadership and political will, Rwanda has made impressive improvements in maternal and child health with the decrease of under-five mortality from 152 per 1000 live births in 2005 to 50 per 1000 live births in 2014 [8, 9]. Additionally, Rwanda specifically emphasized improving the quality of care provided in the newborn period and developed a fully revised National Neonatal Protocol for hospital-based care in 2015 [10]. Despite these achievements, there was no systematic approach to follow and support vulnerable children who remained at developmental risk after surviving the early neonatal period, for example those born premature or at low birth weight [3, 11]. Children with such perinatal risk factors are at increased risk of medical complications, growth failure [12], developmental delay [3], and death [13]. Regular, systematic and ongoing monitoring allows early detection of health, growth, and developmental challenges and subsequently appropriate and timely intervention [14–16]. However, very few models for high-risk children have been tested in sub-Saharan Africa [17].

In 2014, the Rwandan Ministry of Health (MOH), in collaboration with Partners In Health/Inshuti Mu Buzima (PIH/IMB), launched Rwanda’s first Pediatric Development Clinic (PDC) with the overall goal of providing interdisciplinary medical, nutritional, and developmental assessment and intervention in a non-specialist setting to infants and children at high risk for developmental delay [18]. The PDC serves children with premature birth and low birth weight or other perinatal complications, suspected genetic syndromes, and neurodevelopment impairments. However, retention in longitudinal care has proven challenging for health care services in rural African settings [19, 20]. In this study, we assessed PDC patient retention at 12 months post-referral into PDC and factors associated with retention in care.

## Methods

### Study setting and intervention:

We conducted a retrospective cohort study of children enrolled in PDC program between 1April 2014 and 31 March 2015, which was the PDCs first year of operation. Data on visits of children enrolled in this first year were extracted through 31 March 2016 to assess retention at 12 months. Each child was followed for his or her first 12 months in PDC care. This study was conducted in the Rwinkwavu District Hospital (RDH) catchment area in rural Kayonza District, Eastern Province, Rwanda. The catchment area includes eight health centers under RDH supervision, serving a population of about 200,000 [21]. RDH is a MOH public institution that has received support from PIH/IMB since 2005. The PDC was started in April 2014 at RDH and has since been decentralized to two of Southern Kayonza’s eight health centers in August 2014 and two additional health centers in June 2015. During the study time period, an average of 450 deliveries per month occurred in RDH catchment area with about 39 newborns admitted to the neonatal unit each month – it is estimated that about half the these newborns would be eligible for PDC if discharged alive.

PDC aims to improve health outcomes for high-risk children under five years by providing medical, nutritional, and developmental support. The PDC clinic implementation is described in depth in Ngabireyimana et al. (2017), however, a brief description follows. At each visit, caregivers participate in a morning group education session followed by individual consultations with an assessment of the child’s health status, including an assessment of danger signs and vitals, completed by a trained nurse under a General Practitioner’s supervision. Children are treated or referred for specialist care according to the results of assessment. Nutritional support includes growth monitoring, feeding assessment and counseling on breastfeeding and nutrition. Food packages are provided to children whose mothers meet established criteria, including inability to produce sufficient breast milk or those whose social screening documents showed inability to provide adequate nutrition. Infant formula with teaching and safe preparation kits is provided for those infants meeting defined medical therapeutic criteria. Support to optimize child development, which includes regular developmental monitoring using the Ages and Stages Questionnaires [22], individual parent counseling and clinic-based group sessions on child developmental topics, is provided to all children at each visit. Play and communication counseling materials were developed for use in the clinic based on an expanded form of Care for Child Development materials [23]. Condition-specific follow-up is also provided as needed for each child, including kangaroo mother care follow-up for preterm and infants born under 2000 g. Transport reimbursements are provided in cash at each visit to eligible patients, based on nurse and social worker assessment of the caregiver’s ability to pay, to reduce barriers to accessing care for those meeting pre-established criteria per social worker evaluation. Home visits for additional family counseling are conducted weekly by PDC staff to the most vulnerable children as identified by nurses and social workers in the weekly clinic assessments. In addition, community health workers are requested to conduct follow-up home visits with patients who are not making routine appointments.

Children are referred to the PDC either from RDH departments such as Neonatology, Maternity, and Pediatrics or a health center in RDH catchment area, with occasional cases referred from other health facilities outside the RDH catchment area or self-referrals from the community. Children are eligible to enroll in PDC if they have one or more of the following medical conditions: prematurity (< 37 weeks of gestational age or by clinician determination), birth weight under 2000 g, hypoxic ischemic encephalopathy (HIE), cleft lip/palate, hydrocephalus, significant developmental delays, suspected trisomy 21 and/or other suspected genetic syndromes. Eligibility is often determined by a doctor at the point of referral to PDC; in cases of developmental delay, there are no specific diagnostic criteria. However, children who are significantly behind on developmental milestones are often referred by hospital pediatrics wards, health centers, or by self-referral from the community. Referred children are enrolled at the nearest health facility with a PDC for regular follow-up. Follow-up visits are scheduled based on the child’s age and specific medical condition. Individual patient data from each visit is recorded on a paper form and then entered into an Electronic Medical Record (EMR) system.

### Data collection:

Data were extracted from PDC patient charts as well as the EMR for patients who enrolled between 1 April 2014 and 31 March 2015; data on these patient’s visits were then extracted through 31 March 2016 to assess retention at 12 months. Data collected included baseline demographics on children and their primary caretakers, baseline clinical information, social supports received, details of PDC services delivered at each visit and retention outcomes at 12 months. Paper charts were reviewed by trained data collectors. Data quality audits and supervision of data validation were conducted by a research assistant. Crosschecking between EMR data and paper-based data was conducted for key indicators, and identified errors were corrected immediately with recommendations given to improve data quality.

### Measures

Our primary outcome measure was retention in care, defined as a visit within 90 days before or after the 12 month date following the child’s referral date into PDC. Children who were documented to have died or transferred out (discharged or relocated outside the catchment area) of the program were not considered lost to follow-up (LTFU). Period of referral to PDC was defined as a binary variable of two six-month periods (April–September 2014 and October 2014–March 2015).

Gestational age was categorized into four groups: term (37+ weeks), moderate/late preterm (32–37 weeks), very preterm (28–31 weeks) and extremely preterm (less than 28 weeks). Birth weight was collected as a continuous variable and divided into four categories: normal weight (≥2500 g), low birth weight (LBW, 1500–2499 g), very low birth weight (VLBW, 1000–1499 g) and extremely low birth weight (ELBW, < 1000 g).

Diagnosis or reason of referral included all PDC eligibility criteria in addition to children referred for other reasons. Children who were diagnosed with more than one condition were categorized as “diagnosed with multiple conditions” and also counted within each specific condition for which they were diagnosed. We defined a separate variable, “diagnosed with any other conditions”, as any diagnosis that did not include preterm, low birth weight, or HIE due to the small number of children presenting with these other conditions. Socio-economic status was defined as binary variable of “qualifies for government support” to identify the poorest households in Rwanda versus “does not qualify” based on the Rwandan system of *Ubudehe*. *Ubudehe* is a measure of socio-economic status unique to Rwanda that serves as a community-based poverty ranking system; at the time of study there were six wealth categories in *Ubudehe* and the poorest two categories qualified for government support for free health insurance and other social protection services [24].

Social support was defined as provision of conditional cash transfers by the PDC to reimburse the costs of transport to the clinic, conditional food transfers in the form of food packages (either for breastfeeding mothers or as complementary feeding for children over age six months), or a follow-up home visit by community health workers for complicated cases. Infant formula with hygienic preparation kits were provided to infants who met defined medical therapeutic criteria.

### Analysis

We provide descriptive analysis of the patient population, including frequencies, medians and interquartile ranges (IQR). We used Fisher’s exact tests to identify factors associated with retention and LTFU. All factors significant in bivariate analysis at *p* < 0.20 were included in the multivariate analyses. Factors were assessed for collinearity prior to inclusion in the model. Multivariable logistic regression was used to build the final model using backward stepwise procedures. All factors significant at α = 0.05 were retained in the final model.

## Results

From April 2014 to March 2015, 228 patients enrolled in the PDC program; 132 (57.9%) were female and 94 (41.2%) were male (Table 1). Prematurity/low birth weight (62.6%, *n* = 142 out of 227) and HIE (34.5%, *n* = 78 out of 226) were the most frequent reasons for referral. We found that 70.6% of primary caretakers were female (*n* = 161 out of 228), 85% were married or cohabitating (*n* = 195 of 228), and 12.7% had no formal education (*n* = 29 of 228).

**Table 1:** Descriptive characteristics of Pediatric Development Clinic patients and caretakers

	Total (*N* = 228)	
	*N*	%
Child Characteristics		
Gender		
Female	132	57.9
Male	94	41.2
Missing	2	0.9
Age at the first visit (months)		
< 1	83	36.4
1–5	79	34.7
6–11	11	4.8
12+	21	9.2
Missing		
Gestational age at birth (weeks)		
Extremely preterm (< 28)	7	3.1
Very preterm (28–32)	18	7.9
Moderate/late (32–37)	63	27.6
Term (37+)	73	32.0
Missing	67	29.4
Birth weight (grams)		
Normal (> 2500)	66	29.0
Low (1500–2499)	75	32.9
Very Low (1000–1499)	35	15.4
Extremely Low (< 1000)	2	0.9
Missing	50	21.9
Diagnosis (Reason for referral)^**1**^		
PT/LBW (*N* = 227)	142	62.6
HIE (*N* = 226)	78	34.5
Trisomy 21 (*N* = 210)	6	2.9
Cleft palate (N = 210)	4	1.9
Hydrocephalus (N = 210)	3	1.4
Other delay (N = 210)	9	4.3
Other reasons (N = 210)	11	5.2
Diagnosed with multiple conditions (N = 227)	29	12.8
Caretaker Characteristics		
Age (years)		
15–19	12	5.3
20–24	67	29.4
25–34	86	37.7
35–44	39	17.1
44+	4	1.8
Missing	20	8.8
Relationship with the child		
Mother	161	70.6
Father	2	0.9
Grandmother	2	0.9
Missing	63	27.6
Level of education completed		
No education	29	12.7
Some primary school	70	39.7
Primary school	69	30.3
Secondary or higher	8	3.5
Missing	52	22.8
Marital status		
Married or cohabitating	195	85.5
Single, widowed, or divorced	19	8.3
Missing	14	6.1
Socioeconomic status		
Does not qualify for government support	109	47.8
Qualifies for government support	21	9.2
Missing	98	43.0
Number of other dependents in home		
None	20	8.8
1–3 children	109	47.8
4–5 children	29	12.7
6+ children	16	7.0
Missing	54	23.7

*PT/LBW* preterm/low birth weight, *HIE* hypoxic ischemic encephalopathy

^1^Multiple diagnoses per patient were present

Seventy-five percent of children were referred from the hospital (*n* = 172 of 228) and 60.1 % of children were enrolled during the first six months of implementing the PDC program (*n* = 137 of 228) (Table 2). The median days between referral and intake was 9 (IQR: 3–15) and the median number of visits per child in 12 months was 7 (IQR: 5–9). Almost half (47.0%, *n* = 99 of 211) of patients received some form of conditional cash or food transfer from the PDC (including therapeutic formula) in their first 12 months of care. Four percent of the children received an additional home visit by a community health worker (*n* = 10 of 228).

**Table 2:** Pediatric Development Clinic visits in first 12 months of care

	Total (*N* = 228)	
	*n*	%
Source of referral		
Hospital	172	75.4
Health Centers	17	7.5
Other	6	2.6
Missing	33	14.5
Health Facility at first visit		
Rwinkwavu	133	58.3
Kabarondo	76	33.3
Ndego	19	8.3
Household’s nearest health center has a PDC		
No	56	24.6
Yes	168	73.7
Missing	4	1.8
Period of referral to PDC		
April 2014–September 2014	137	60.1
October 2014 – March 2015	91	39.9
Patient mode of transport to PDC		
Walking	90	39.5
Motorbike	76	33.3
Mini bus	38	16.7
Other	15	6.6
Missing	9	4.0
Patient transferred between PDCs		
No	194	85.1
Yes	34	14.9
Number of visits in 12 months, median (IQR)	7	(5, 9)
Patient ever received food packages		
No	163	71.5
Yes	47	20.6
Missing	18	7.9
Patient ever received infant formula		
No	200	87.7
Yes	10	4.4
Missing	18	7.9
Patient ever received transport reimbursement		
No	118	51.8
Yes	93	40.8
Missing	17	7.5
Patient ever received a CHW home visit		
No	200	87.7
Yes	10	4.4
Missing	18	7.9

*PDC* Pediatric Development Clinic, *IQR* Interquartile range, *CHW* Community Health Worker

Out of 228 children, 147 (64.5%) were retained in care after one year, 74 (32.5%) were LTFU, four (1.8%) died and three (1.3%) were transferred out of the program (Table 3). In the unadjusted analysis, male sex (*p* = 0.189) and having more children at home (*p* = 0.027) were both socio-demographic factors associated with increased retention (Table 4). Having a diagnosis other than preterm/low birth weight or HIE (“other diagnosis” such as trisomy 21, cleft lip/palate, etc.) was associated with lower retention (*p* = 0.024). The health facility of first visit (*p* = 0.006), having a PDC in the nearest health facility (*p* = 0.136), and period of referral to PDC (*p* = 0.006) were associated with increased retention in care at 12 months. Social support was significantly associated with retention in care with 100% of children who received food packages (*n* = 47, *p* < 0.001), infant formula (*n* = 10, *p* = 0.035), transport fees (*n* = 90, p < 0.001), and community health worker home visits (*n* = 10, *p* = 0.035).

**Table 3:** Patient retention status at 12 months by diagnosis

	In care		LTFU		Died		Transferred Out	
	*N*	%	*n*	%	*n*	%	*n*	%
All children (N = 228)	147	64.5	74	32.5	4	1.8	3	1.3
Diagnosed as PT/LBW (N = 142)	95	66.9	42	29.6	2	1.4	3	2.1
Diagnosed with HIE (N = 78)	54	69.2	22	28.2	1	1.3	1	1.3
Diagnosed with Trisomy 21 (*N* = 6)	1	16.7	5	83.3	0	0.0	0	0.0
Diagnosed with cleft lip/palate (N = 4)	2	50.0	2	50.0	0	0.0	0	0.0
Diagnosed with hydrocephalus (*N* = 3)	2	66.7	1	33.3	0	0.0	0	0.0
Diagnosed with other developmental delays (N = 9)	4	44.4	5	55.6	0	0.0	0	0.0
Diagnosed with other condition (*N* = 11)	6	54.6	4	36.4	1	9.1	0	0.0
Diagnosed with multiple conditions (N = 29)	19	65.5	9	31.0	1	3.5	0	0.0

*LTFU* lost to follow up, *PT/LBW* preterm/low-birth weight, *HIE* hypoxic ischemic encephalopathy

**Table 4:** Bivariate associations with retention to care at 12 months

	Retained (*n* = 147, 66.5%)		LTFU (*n* = 74, 33.5%)		*P* value
	*n*	%	*n*	%	
Gender (*N* = 219)					
Female	81	63.3	47	36.7	0.189
Male	66	72.5	25	27.5	
Age at the first visit (months) (*N* = 203)					
< 1	61	74.4	21	25.6	0.570
1–5	54	70.1	23	29.9	
6–11	6	54.6	5	45.5	
12+	23	69.7	10	30.3	
Gestational age in weeks at birth (weeks) (*N* = 156)					
Extremely preterm (< 28)	4	57.1	3	42.9	0.861
Very preterm (28–32)	12	75.0	4	25.0	
Moderate/late (32–37)	40	65.6	21	34.4	
Term (37+)	48	66.7	24	33.3	
Birth weight (grams) (*N* = 171)					
Normal (> 2500)	43	67.2	21	32.8	0.386
Low (1500–2499)	46	63.9	26	36.1	
Very Low (1000–1499)	26	78.8	7	21.2	
Extremely Low (< 1000)	1	50.0	1	50.0	
Diagnosed with PT/LBW (*N* = 220)					
No	52	62.7	31	37.4	0.376
Yes	95	69.3	42	30.7	
Diagnosed with HIE (N = 219)					
No	93	65.0	50	35.0	0.450
Yes	54	71.1	22	29.0	
Diagnosed with any other conditions (*N* = 220)					
No	132	69.8	57	30.2	0.024
Yes	15	48.4	16	51.6	
Diagnosed with multiple conditions (N = 220)					
No	128	66.7	64	33.3	> 0.999
Yes	19	67.9	9	32.1	
Age of the primary caretaker (years) (*N* = 201)					
15–19	9	81.8	2	18.2	0.595
20–24	45	70.3	19	29.7	
25–34	55	65.5	29	34.5	
35–44	22	57.9	16	42.1	
45+	3	75.0	1	25.0	
Caretakers relationship with the child (*N* = 159)					
Mother	104	67.1	51	32.9	> 0.999
Father	2	100.0	0	0.0	
Grandmother	1	50.0	1	50.0	
Caretaker’s level of education (N = 172)					
No education	23	79.3	6	20.7	0.387
No formal education completed	48	68.6	21	30.4	
Primary school completed	49	74.2	17	25.7	
Secondary or higher completed	4	50.0	4	50.0	
Caretaker’s marital status (*N* = 207)					
Married or cohabitating	127	67.2	62	32.8	0.608
Single, widowed, or divorced	11	61.1	7	38.9	
Household socioeconomic status (*N* = 128)					
Does not qualify for government support	74	69.2	33	30.8	0.315
Qualifies for government support	12	57.1	9	42.9	
Number of other dependents in home (*N* = 170)					
None	10	50.0	10	50.0	0.027
1–3 children	81	75.0	27	25.0	
4–5 children	18	69.2	8	30.8	
6+ children	15	93.8	1	6.3	
Source of referral (*N* = 190)					
Hospital	107	64.1	60	35.9	0.416
Health Centers	13	76.5	4	23.5	
Other	5	83.3	1	16.7	
Health Facility of first visit (*N* = 221)					
Rwinkwavu	77	60.6	50	39.4	0.006
Kabarondo	52	69.3	23	30.7	
Ndego	18	94.7	1	5.3	
Household’s nearest health center has a PDC (*N* = 217)					
No	32	58.2	23	41.8	0.136
Yes	113	69.8	49	30.3	
Period of referral to PDC (N = 221)					
April 2014–September 2014	79	59.4	54	40.6	0.006
October 2014 – March 2015	68	77.3	20	22.7	
Patient mode of transport to PDC (*N* = 212)					
Walking	58	67.4	28	32.6	0.832
Motorbike	47	63.5	27	36.5	
Minibus	26	70.3	11	29.7	
Other	9	60.0	6	40.0	
Patient transferred between PDCs (N = 221)					
No	121	64.7	66	35.3	0.236
Yes	26	76.5	8	23.5	
Patient ever received food packages (*N* = 205)					
No	97	61.4	61	38.6	< 0.001
Yes	47	100.0	0	0.0	
Patient ever received infant formula (N = 205)					
No	134	68.7	61	31.3	0.035
Yes	10	100.0	0	0.0	
Patient ever received transport fees (*N* = 206)					
No	55	47.4	61	52.6	< 0.001
Yes	90	100.0	0	0.0	
Patient ever received a CHW home visit (N = 205)					
No	134	68.7	61	31.3	0.035
Yes	10	100.0	0	0.0	

*LTFU* lost to follow up, *PT/LBW* preterm/low-birth weight, *HIE* hypoxic ischemic encephalopathy, *PDC* Pediatric Development Clinic, *CHW* Community Health Worker

When adjusting for covariates, the period of referral (odds ratio (OR): 2.56; 95% confidence interval (CI): 1.36, 4.80, *p* = 0.004) was associated with increased retention in care (Table 5), and “other diagnosis” continued to be associated with decreased retention in care at 12 months (OR: 0.34, 95% CI: 0.15, 0.76, *p* = 0.009) compared to children who had either preterm/low birth weight or HIE. We were unable to assess social support in the adjusted model as receipt of support completely predicted retention in care; site of first visit was also not included in the full model due to collinearity with the period of referral to PDC.

**Table 5:** Multivariate analysis of predictors of retention in Pediatric Development Clinic at 12 months

	Full Model			Reduced Model		
	OR	95%CI	*P* value	OR	95%CI	*P* value
Child sex						
Female	Ref					
Male	1.48	(0.79, 2.74)	0.219			
Diagnosed with any other conditions^1^						
No	Ref			Ref		
Yes	0.31	(0.14, 0.70)	0.005	0.34	(0.15, 0.76)	0.009
Household’s nearest health center has a PDC						
No	Ref					
Yes	1.70	(0.87, 3.31)	0.12			
Period of referral to PDC						
April 2014–September 2014	Ref			Ref		
October 2014 – March 2015	2.50	(1.32, 4.74)	0.005	2.56	(1.36, 4.80)	0.004

*PDC* Pediatric Development Clinic

^1^Diagnosed with any other condition includes diagnosis of Trisomy 21, cleft palate, hydrocephalus, other delay, or other reasons

## Discussion

In our study, we found 64.5% retention for patients referred to PDC in the first 12 months, which is promising for a newly implemented program. However, studies on HIV treatment retention in infants in low- and middle-income countries show a higher retention [19, 25]. We also observed a low documented mortality rate for children enrolled in the PDC compared to other studies in developing countries for patient groups of a profile similar to the majority of PDC patients, including for children with very low birth weight [3] and birth asphyxia [26]. We assume that the PDC program was beneficial for these high-risk infants, however an evaluation comparing outcomes to a baseline conducted prior to the implementation of the program in the same population is still ongoing.

Our study showed that receipt of social support completely predicted retention in care. This result is unsurprising as the PDC serves a rural population with very limited resources with a quarter of the population living in poverty [27] and these supports may serve as a financial incentive for participation in the PDC program. The provided social support helps to remove financial barriers to participation in care, and we contend, is a critical component to support the health and development of these children. For example, provision of breastfeeding support, nutritional counseling, and infant formula when medically necessary is extremely important to the brain growth and development of premature children who have catch-up growth needs and may have feeding difficulties. Perceived (and actual) improvement in a child’s growth would certainly provide encouragement to the child’s caretakers to return to PDC. Social support was also found as predictor of good retention and good outcomes for an HIV clinic program for adults in a rural poor setting area [28]. In addition, partnering home visits with pediatric care as we have done has been shown to be a strong predictor of retention. In a study of a home visiting program in the United States, home-visited mothers kept pediatric appointments 10 times more than those who did not receive home visits [29].

The findings of an increase in retention in care in the second six-month period of referral in the PDC’s first year of operation might be related to the increased awareness of the program importance over time; the more the population became aware of the PDC program, the more the retention in care increased. This finding might also be attributed to improved quality of care provision as providers gained more experience and iterative learning and improvement over time, particularly around identifying children who were missing visits. In addition, it was in the second six-month period of operation where the four decentralized health center clinics were all fully operational for the full time period, which may have eased access to care and contributed to greater retention.

Our analysis showed a relationship between less-common conditions such as Trisomy 21, cleft palate, hydrocephalus and other developmental delays and increased LTFU. This could be a result of a few different factors. First, stigma or misperceptions in the community of these conditions could deter care seeking and encourage a preference for keeping children with such conditions a secret [30]. Research in Malawi showed the caretakers of children with intellectual disability require supports to address mental health issues that arise due to elevated stress and stigma experienced when caring for these children [31]. Also, there could be some potential discouragement among caretakers as it may take more time to see change in children experiencing more pervasive developmental delays when compared to prematurity conditions that can develop quite normally with appropriate supports. This might also be related to the unique management of some of these conditions, which include surgical repair for cleft lip and palate. Once managed there may have been no need to continue with close follow up of these children. Further, conditions such as hydrocephalus require referrals for neurosurgery evaluation. LTFU may occur in the process of this transfer to a referral facility; better understanding of continuity of care following referrals is an important area for further investigation in this novel program. The sample size of those conditions is too small in the program to draw definitive conclusions and further studies are needed to better understand the trajectory of these children in care.

The findings of our study need to be taken in context within some limitations. As our study used routinely collected data from patient charts and files, we found significant levels of missing data. Additionally, because we used routinely collected program data, information on some individual factors that might influence retention in care were not available. Respondents are not always able to provide information on variables such as gestational age due to challenges in determining gestational age [32] and *Ubudehe* status, which has been reported as unknown by a quarter of people in large national surveys [33] and further contributes to missing data. However, important information was provided despite these data limitations. Another limitation is generalizability of our findings; because PDC is a pilot program only operational at one district hospital and four health centers in rural Southern Kayonza District, the findings may not be generalizable to other settings. Nevertheless, this study can provide important information to program implementers to ensure high retention and help inform replication of the PDC program in other areas in Rwanda, other programs in rural African settings, or other countries with low resources.

The results from this study are heartening and highlight both the viability of providing longitudinal care through a program reaching a previously-underserved population of children in a rural, resource-limited African setting, as well as the importance of social support in retaining these at-risk children in care over the long term. While studies are underway to assess other factors related to feasibility of the PDC program like costs, acceptability and ability to self sustain as well as to understand the long-term impact of the PDC care on the health and developmental outcomes of these high-risk children, this program can serve as an example in other similar settings.

## Conclusions

The PDC model implemented in rural Rwanda demonstrates promising retention rates at 12 months for a new clinic and low rates of documented mortality in this high-risk population of very young children. This model of integrated and holistic follow-up could contribute to strong retention in other early childhood development programs and may improve future outcomes of children at high risk for development delay in resource-limited settings.

## Abbreviations

HIE: hypoxic ischemic encephalopathy
LTFU: Loss to follow-up
MOH: Ministry of Health
PDC: Pediatric Development Clinic
PIH/IMB: Partners In Health/Inshuti Mu Buzima
RDH: Rwinkwavu District Hospital
